# Perceptions and conducts of oral and maxillofacial surgeons during the COVID-19 pandemic: A qualitative study

**DOI:** 10.1371/journal.pone.0286853

**Published:** 2023-06-09

**Authors:** Ricardo de Oliveira Corrêa, José Alcides Almeida de Arruda, Amanda Isabela Firmino Gomes, Evandro Guimarães Aguiar, Efigênia Ferreira e Ferreira, Cláudia Silami de Magalhães, Amália Moreno

**Affiliations:** 1 Department of Oral Surgery, Pathology and Clinical Dentistry, School of Dentistry, Universidade Federal de Minas Gerais, Belo Horizonte, Minas Gerais, Brazil; 2 Department of Social and Preventive Dentistry, School of Dentistry, Universidade Federal de Minas Gerais, Belo Horizonte, Minas Gerais, Brazil; 3 Department of Restorative Dentistry, School of Dentistry, Universidade Federal de Minas Gerais, Belo Horizonte, Minas Gerais, Brazil; University of Verona, ITALY

## Abstract

Oral and maxillofacial surgeons are among the frontline healthcare workers and are classified as a high-risk group for COVID-19 infection; however, it has not yet been defined how these professionals were impacted. The aim of this study was to explore the conducts and perceptions of oral and maxillofacial surgeons during the COVID-19 pandemic in Brazil. Nine individuals, mean age 34.8 years, 66.6% men, were included in the study. A semi-structured interview with a qualitative approach was applied to professionals belonging to a messaging application group (WhatsApp). Content analysis was performed in the light of Hellerian theory in its daily theoretical framework for the interpretation of the memories reported by the participants. Four themes were identified. The lack of knowledge about COVID-19 and the fear of being contaminated during care were the main factors responsible for changes in the professionals’ work routine. An important point was the collective reflection of the participants about the increase in biosafety barriers, which ensured a greater sense of security. The need for social isolation to contain the virus was also described. As a result, there was a great distance between professionals and their families, which generated high levels of anxiety in the former. Repetitive reports of slowness and reduced attendance directly related to financial loss and aggravated stress were also highlighted. The findings of this study reveal that oral and maxillofacial surgeons had their professional-personal axis affected in terms of daily habits, family life and financial strain, aspects that were responsible for impacting stress and anxiety levels.

## Introduction

The outbreak of the SARS-CoV-2 virus set up an unprecedented pandemic, causing the collapse of healthcare systems worldwide due to the high number of hospitalizations and deaths [1]. Oral health professionals occupy one of the highest risk positions for disease contagion [2]. These professionals, as well as their patients, are constantly exposed to numerous pathogenic microorganisms, including viruses present in the respiratory tract and oral cavity. Hence, those who work in a dental office are at risk of coronavirus 19 (COVID-19) infection due to the specificity of some procedures, involving proximity and face-to-face communication with the patient and invariably generating frequent exposure to saliva, blood and other body fluids, in addition to the handling of instruments that can generate aerosols [3]. Likewise, the specialty of oral and maxillofacial surgery is particularly considered to involve a high risk of disease transmission due to the large generation of aerosols during surgical interventions, proximity to the patient during surgery and especially to the work environment–often in hospitals [4,5].

Although relevant, several recently published quantitative studies on the impact of COVID-19 on the practice of oral and maxillofacial surgery have left a gap to be investigated with respect to the focus on the perception and behavior of these professionals when experiencing the current scenario [6–9]. Thus, a qualitative approach can support evidence for quantitative investigations in addition to promoting useful and meaningful data about the spectrum of experiences of professionals working in the field of oral and maxillofacial surgery, from the decision-making about different treatments to the biosafety protocols to be adopted. Furthermore, qualitative studies allow individuals to describe their experiences as proposed by the theoretical framework of the Agnes Heller theory of everyday life [10], which proposes the capacity of an individual to critically assess a question through a more conscious, reflective and secure argumentation [10]. These studies may also address multiple aspects, such as providing information and experiences in the workplace [11].

Therefore, the objective of the present study was to explore the perceptions and behaviors of oral and maxillofacial surgeons facing the COVID-19 pandemic. A qualitative approach in this current scenario is not only relevant, but necessary, as it can lead to a more in-depth knowledge of the new routine experienced by that this profile of professionals.

## Materials and methods

This is a study with a qualitative approach whose saturation was employed for satisfaction in data production, an approach that justifies the reduced number of participants for this style of analysis, maintaining internal and external validity. The present study has been reported according to the statement of COREQ (COnsolidated Criteria for REporting Qualitative research) [12]. The study was approved by the Ethics Committee of Universidade Federal de Minas Gerais (#40839620.80000.5149) and the participants gave written informed consent for the publication of the study in agreement with the Declaration of Helsinki.

Non-probabilistic convenience sampling was employed for data collection according to the number of professionals who met the study inclusion criteria. The first phase consisted of the selection of oral and maxillofacial surgeons belonging to a group of messaging application named WhatsApp (Facebook Inc., Menlo Park, CA, USA). This group is composed exclusively of oral and maxillofacial surgeons with a unique interest in establishing dialogue, exchanging experiences and doubts among these professionals who work in hospitals and outpatient clinics in Minas Gerais, Brazil. A key informant who was familiar with these professionals invited each one of them to participate in the study via a text message. At this point, all subjects were fully informed about the purpose of the study and what their involvement would entail. We offered no economic incentives to the participants. The inclusion criteria were those professionals who showed complete interest in participating in this study. Exclusion criteria imposed were participants who did not complete all interviews and dropped out during the interview.

In the second phase, the Microsoft Teams platform (Microsoft, Redmond, Washington, USA) was used to carry out the recorded interviews, which were later transcribed. For the qualitative interviews, a guiding script was used that allowed the participants to talk freely about relevant and representative events of the “new normal” in which they lived with the COVID-19 pandemic. The interviews did not have a pre-established average duration and all started with the same triggering question: “Tell me about being an oral and maxillofacial surgeon, from the treatments performed to the difficulties involved”. Some other approaches related to the work of contemporary dentistry, adaptation to the service and, at the end, to personal and professional impressions and contributions to the history of the disease COVID-19 were also raised. An interview was carried out by an author (R.O.C.) with a volunteer belonging to the same study sample in order to test the script and train the interviewer. However, this (pilot) interview was not included in the study.

After this initial phase and adaptation of the methodology, the interviews of the participants were conducted by the same interviewer (R.O.C.) after scheduling an appointment respecting the availability of time of each participant. The response saturation strategy (when no new data were identified or the data already detected were rich and in-depth) was used to complete the interviews and the number of respondents. All interviews were validated with the participants through a synthesis of the content presented by the interviewer right after the end of the interview. These interviews were recorded in full, transcribed and finally analyzed by two calibrated authors (R.O.C. and A.M.). The names of the professionals were omitted and the interviewees were represented by the term “surgeon” followed by a number. A field diary was carried out, containing notes about the interview such as impressions, description of behaviors, physical space, and comments/reflections, among others. The qualitative data obtained were stored in memos. The survey lasted for a period of approximately 30 days (April 14 to May 7, 2021). The material recorded from the qualitative interviews with a semi-structured script was transcribed into text using the Word program (Microsoft, Redmond, Washington, USA) and submitted to various readings to obtain a deeper understanding of the reports.

We followed Charmaz’s Grounded Theory approach since it is appropriate for focusing on the interpretative understanding of experiences, giving voice to the participants themselves [13]. In the analyses, after identifying the nuclei of meaning, the text of each one was condensed in order to identify the essence of the statements. Through this process, codes, sub-themes and themes were created. Codes are units of meaning that allow data to be thought about and translated, and their set determines a sub-theme. Sub-themes, when similar in meaning, can determine a theme [14]. Thus, the analysis allowed the identification of sub-themes and themes of the study. From the perspective of Agnes Heller’s theory of everyday life, the analysis was conducted in order to understand the meaning of the routines, biosafety care, as well as social and family life of the professionals [11,15].

## Results

Data were collected in individual interviews, resulting in a total of nine participants. Mean age was 34.8 years, ranging from 30 to 41 years, and 66.6% (*n* = 6) were men. The mean experience time of oral and maxillofacial surgeons was 5.3 (±2.5) years (range: 2–10 years). The duration of each interview ranged from 10 to 34 minutes. None of the study subjects withdrew from participating during the interviews. Four main themes emerged from the data after thematic analysis: (I) perception of professionals about the specialty of oral and maxillofacial surgery, (II) biosafety, (III) psychosocial aspect of professionals, and (IV) impact on attendance. There was no premeditation of the themes and their definitions were in accordance with the data analysis, as detailed in **Table 1**.

**Table 1:** Data analysis themes and sub-themes.

Themes	Sub-themes
**Perception of professionals about the specialty or oral and maxillofacial surgery**	• Wide presence in the labor market • Knowledge, responsibility and dedication • Difficulties faced by the hospital work team • Importance of the dentist in the prevention and cure of diseases
**Biosafety**	• Attention to biosafety and procedures at work • Face shields incorporated into personal protection equipment
**Psychosocial aspect of professionals**	• Stress, anxiety and insecurity • Fear of contamination in the work field • Impact on interpersonal and family relationships • Appreciation of family and friends • New habits on arrival at home
**Impact on attendance**	• Decrease versus no change/increase in patient demand • Urgent care during the COVID-19 pandemic • Patients afraid to be treated • Financial commitment

### Perceptions of professionals about the specialty of oral and maxillofacial surgery

In this major theme, the participants reported about their motivational aspirations regarding the option of pursuing a career in the specialty of oral and maxillofacial surgery. In addition, they contextualized the field of action in the labor market, pointing out the different universes of hospital/outpatient action, multiple jobs, or just the vectors of public or private services. They also visualized their feelings related to the difficulty in becoming specialists (due to the demands of a lot of knowledge and personal dedication), and how the performance of their functions was peculiar and necessary in the context of preventive and curative dentistry.

#### Wide presence in the labor market

Some participants considered the specialty of oral and maxillofacial surgery to involve more complete training in dentistry, preparing professionals to work in environments such as the private clinics and hospitals.

*“I work in the two modalities. And I think it is equally important to see patients in the office for minor oral surgeries and to work with patients in hospital clinics for major oral surgeries! A complete oral and maxillofacial surgeon works in all areas! So, I work in the two modalities.”*

(Surgeon 1)

*“So, we are inserted both in the hospital context and in the outpatient clinic context and I think that our presence and our function are of primordial importance for the wellbeing of the patients.”*

(Surgeon 2)

*“Oral and maxillofacial surgery is an extremely wide area of dentistry and, in my opinion, it is important because it treats not only the teeth but also the dentofacial deformities, as well as their oral manifestations and facial trauma.”*

(Surgeon 3)

In the hospital environment, the work expands the range of activities of the dentist, including surgical treatments of impacted teeth, biopsies/minor surgeries, moderate/severe odontogenic infections, and acting on the face, such as facial traumas and orthognathic surgeries.

*“I do surgeries of low complexity, that is, outpatient procedures here in the CEO (Center of Dental Specialties) of the municipality, and I also have a private practice in some offices where I do outpatient surgeries, and also private procedures in the hospital area.”*

(Surgeon 4)

*“I work and act on an outpatient basis in the office for minor oral surgeries, the extraction of impacted teeth, and some oral biopsies. And I also work in the hospital area, which is what makes the surgeon’s heart really flutter; I work with facial trauma, with accident victims, with orofacial lesions, and with orthognathic surgeries.”*

(Surgeon 5)

#### Knowledge, responsibility and dedication

Most participants indicated that oral and maxillofacial surgery is a field that requires multiple knowledge, dedication, responsibility, and persistence for the professionals to achieve their work/performance goals and, thus, there are difficulties in entering the career.

*“The surgical area and the oral and maxillofacial area, in particular, require ample knowledge on our part so that we may feel safe in managing the patients and their diseases.”*

(Surgeon 4)

*“…it is a slightly more complex field for you to enter, you must devote more time to it (at times the tone of his speech changes, demarcating the narrative), you must be patient, join the team and, once you are in, wait to be invited to the operating table. This takes some time (a positive head nod)! So, you will have to set aside some days of the week to deal with this.”*

(Surgeon 6)

*“Within our range of services for patients in the area of dentistry, I see oral and maxillofacial surgery as the most complete. Why? Before I specialized in oral and maxillofacial surgery, I was a general clinical surgeon. So, I know how to counsel a patient, from a simple cleaning to a complex orthognathic surgery or even about a more serious problem such as malignant tumors, when I work with the head and neck surgeon (throughout his speech he makes head movements emphasizing his words).”*

(Surgeon 7)

*“…behind all of this there is a lot of struggling, many shifts, a lot of sacrifice, and many hours when you can’t be with the people you like, with your family and friends, and you face a lot of difficulties.”*

(Surgeon 4)

#### Difficulties faced by the hospital work team

Oral and maxillofacial surgeons have the hospital environment as one of their areas of activity. A certain challenge of the participation of these professionals within the multidisciplinary health team was described.

*“…the great difficulty I face today is acceptance of my participation also within the health areas as a whole (…), recognition by both the medical and the nursing teams.”*

(Surgeon 3)

*“…the demand for this service is great despite the resistance of some directors (shakes his head negatively) and councils, I don’t know why, about giving a definitive place to the dental surgeon so he can take care of the oral health of hospitalized patients.”*

(Surgeon 7)

*“The main difficulties we face are at the hospital level (…); we don’t have much access to hospitals, which are more limited to medical doctors (frowns) and many oral and maxillofacial surgeries are performed in this environment; so, I think the main difficulties are those of our specialty. What I see is a greater difficulty, a greater obstacle to our access to the hospital environment.”*

(Surgeon 1)

#### Importance of the dentist in the prevention and cure of diseases

The participants in this study reported that elective treatments were mostly suspended during the pandemic. This was due to the lack of knowledge about how the virus disseminated and the need to have a health focus on emergencies resulting from the COVID-19 disease itself, in addition to the scarce availability of supplies for the manufacture of hospital and personal protection equipment. Therefore, an important fact reported by the participants was the recognition of the specialty for the cure of diseases of possible negative evolution, potentially causing death.

*“…I also work in this area of odontogenic infections (…) where we do extraoral drainage and we can cure the patients, so that they will not develop more serious conditions requiring an Intensive Care Center. (…) It is an area in which we are able to cover various sectors, not just surgeries (…), but also diagnoses. I can say this is the area that I like best today, where I can deal with the question of prevention of benign and malignant diseases of the oral cavity.”*

(Surgeon 8)

*“…the importance of dental treatment, of preventive treatment, preventive maintenance, and visits to the dentist; this also helps prevent more serious cases of COVID-19 (changes his tone, with emphasis on the end of his speech)!”*

(Surgeon 3)

A striking aspect reported by some of the participants was the clear and necessary perception, by society, of the dentist’s performance within hospital environments as a routine and not in an occasional way. According to their reports, this period of lack of care created a different perspective about the importance of dentistry for the population.

*“…my friend, a physician, told me ‘Surgeon, I don’t know why there is no dentist in the hospital to provide this basic care for the patients regardless of where they are, on a ward, in the Intensive Care Center or the Intensive Care Unit. A female patient looked at me and said: ‘oh doctor, I did not know how valuable a dental treatment was! I only learned about it after I felt pain and I had no place to go, did not know what to do’.”*

(Surgeon 7)

### Biosafety

Biosafety was cited as a decisive factor by all participants in preventing and combating the spread of SARS-CoV-2. They stated that biosafety is indeed a routine, especially in the surgical area, but new and even stricter protocols were incorporated into it in order to create greater protection for the entire team responsible for dealing with COVID-19, as well as for patients at potential risk. The main items added to the vast list of personal protection equipment mentioned by all participants were the N95/PFF2 masks and the face shields.

#### Attention to biosafety and procedures at work

Faced with a pathogen with a high contamination ability, a high mortality rate and a worldwide lack of knowledge about the virus, there was a real need to change biosafety standards. One of the measures most commonly taken was, at the beginning, the stoppage of services. Subsequently, there was greater spacing between consultations, in addition to the use of facial protection barriers.

*“…I also was not quite aware of the idea of a waiting interval between one patient and another; so, as a precaution, I chose to make appointments every 2 hours.”*

(Surgeon 8)

*“…paying attention to the little things that I miss in my daily routine, do you understand? That rush? Ah, I’m not going to do this, I will not pass alcohol between one patient and another, it is only removal of sutures. It is only this, it is only that (…) and then an accident may happen and I will be contaminated or I will contaminate somebody else; so, this type of care came back a little stronger for everybody, especially in dentistry.”*

(Surgeon 4)

*“To reinforce biosafety and mainly the personal protection equipment (…). I often tended to neglect personal protection equipment, a mask, a cap (raises his eyebrows) and sometimes the disinfection of the office itself, things like that. So, I think it ends up leaving or turning the spotlight on this part of biosafety and personal protection equipment.”*

(Surgeon 9)

#### Face shields incorporated into personal protection equipment

For a virus transmitted mainly by droplets and aerosols, there was a need to develop devices capable of offering protection to those closest to potentially infected people. One of the main personal protection equipment recommended was the face shield.

*“But, as I said, a face shield is something that makes a lot of difference for those who work with blood right in front of their face. I really feel this difference.”*

(Surgeon 8)

*“…I started to look for ways to increase safety for us professionals (…) and to use a face shield (indicates with her hand in position on her face) to reduce the droplets that might fall on my face.”*

(Surgeon 4)

*“I used to wear the eyeglasses and common mask that we use for surgery. But after we see how dirty the face protector is after a simple dental cleaning or restoration or after an endodontic treatment without the use of much spray, there are always some droplets left on the face shield. No way that I will abandon the facial shield. Regardless of the pandemic or not! This was the main point!”*

(Surgeon 7)

### Psychosocial aspect of professionals

Most participants reported a change in their psychological state, partially affecting their mental health. Stress and increased level of anxiety were the items most frequently mentioned by the respondents. Other aspects reported on a smaller scale were fear and uncertainty regarding COVID-19. In addition, all of these sensations caused disturbances in personal well-being, with a really negative impact.

#### Stress, anxiety and insecurity

There was uncertainty due to not knowing whether oral and maxillofacial surgeons were sufficiently protected, whether these professionals had been exposed to the virus or not, whether they could be a vehicle of contamination for family members, among many other concerns. Consequently, the levels of stress and anxiety among them became alarming.

*“No, it had a great impact on wellbeing (opens her eyes, shakes her head, changes her expression completely)! I think that those who say that there was no impact are lying (light smile)!! (…) So, this question of support, of the affection we feel and exchange with people we like (shows with her hands the intensity of her speech). Being unable to do this is very complicated (…) Psychological in every sense (gesticulating, shaking her head, with an afflicted smile and clearly showing the extent of this impact)!!!!”*

(Surgeon 2)

*“…and this really was stressful for me as a person, I worried a lot about whether I was taking the proper care, I watched a lot of lives, I watched a lot of lectures, I read a lot of articles at the beginning of last year to find out if I was really following the full safety protocol, but this created stress!”*

(Surgeon 8)

*“Well, as to my personal well-being, my nature is to be a little anxious. I think this got a little worse during the initial phase of the pandemic. Yes, I must say that, really, I manifested some attitudes that perhaps I would not have manifested during more tranquil and calm times. So, I believe that I had some peaks of anxiety (the participant made this statement with a thoughtful expression, pausing to think before starting to speak and frowning repeatedly throughout his speech)!”*

(Surgeon 5)

#### Fear of contamination in the work field

The working environment of the specialist in oral and maxillofacial surgery, as well as that of other health professionals, was stigmatized, whether it involved an outpatient clinic or office, or a hospital environment. The fear of being infected by the virus during care due to the essential proximity of the airways of patients without a mask, was a major factor. The oral and maxillofacial surgeon was trapped in his/her work environment.

*“And even with the personal protection equipment I felt unsure about running the risk of being contaminated.”*

(Surgeon 4)

*“First, my worry about being contaminated during work of course increased (shows a worried expression), and second my worry about contaminating my relatives who were confined, in particular my mother-in-law!”*

(Surgeon 7)

*“So, I am highly exposed, being already at risk simply by being in contact with a patient and I participate in a surgery where we open up and there is blood and at times an aerosol due to the use of a pen or something like that. So, I’m trying to isolate myself as much as possible from other people because I don’t know up to what point I am protected (moves his hands in a rhythmic way) and if I may put these people at risk.”*

(Surgeon 6)

*“…we work in an environment which is a means of transmission of COVID-19, that is, the oral cavity (…). Even if we follow all the protocols, face shield, N95 mask, use of disposable gowns (quickly looks up), room disinfection and sterilization, we still have fear. Fear to get COVID-19 from the patients and to take it inside our home.”*

(Surgeon 3)

#### Impact on interpersonal and family relationships

In some instances, the participants were found to be intense and aligned when answering that what most affected them was the absence of close family and friends. The lack of affection and support from the family network was decisive in creating an extremely negative and striking situation for everyone. Regarding the strong bonds and affective bonds with family members, especially parents, to keep them safe, the interviewees adopted attitudes of total detachment, in some cases for long periods.

*“I no longer was in contact with my family and I continued working in hospitals, and I think this had a highly emotional impact!”*

(Surgeon 4)

*“…I had to stay away mainly from my parents, you know?! To protect them and also to protect my brother. On a personal level, many family members were deprived of visits and this had a great impact on me!”*

(Surgeon 1)

*“It’s been 4 months since I saw my parents. I gave up seeing them (saddened tone) to avoid any kind of contamination!”*

(Surgeon 6)

#### Appreciation of family and friends

The impossibility of having something that was previously within reach, the absence of routines and social contact, especially family, which previously seemed so commonplace, generated a new meaning in most people. A new trend emerged bringing the pleasure of leisure into the family nucleus and the intimate circles of friends.

*“…I started to learn to appreciate what really matters. To see how important it is for me to have these moments, obviously not right now, but when things return to normal, to be close to the people I like, and not just continue with this madness, with living only for work!”*

(Surgeon 2)

*“From the family point of view, I think this pandemic made me get closer to my children (…), I was able to spend more time with them and this was good for a closer affective relationship with them!”*

(Surgeon 5)

#### New habits on arrival at home

The highly contagious SARS-CoV2 virus has created widespread change in all routines. For example, dressing and undressing has become a risk factor when considering the possibility of bringing such a virus into the home.

*“So, I had to change my routine when getting back home (a worried look) regarding my clothes. I had to devote an exclusive area to my return home from work so that I could take off my clothes to avoid as much as possible to walk around the house wearing my shoes; actually, to circulate as little as possible inside the house wearing the clothes I brought home from the office.”*a

(Surgeon 7)

*“I modified some things. For instance, I no longer enter my house wearing my shoes. I get in through the kitchen door and I go to a little room, a pantry, where I take off the clothes I was wearing at work. I don’t walk anymore inside the house wearing those clothes.”*

(Surgeon 6)

### Impact on attendance

With the onset of the pandemic and the need to contain it, there were changes in the care provided by the participants and in their workplace, with such changes clearly reflecting on job performance. Only one participant did not complain about the changes. Emergency care became routine and the patients themselves abandoned it out of fear. Financial losses resulting from the interruption of services aggravated the stressful situation, a fact reported by several participants.

#### Decrease versus no change/increase in patient demand

In general, the specialty of oral and maxillofacial surgery, when elective, has a predilection for the older portion of the population. In the context of the pandemic, in which older adults were considered a risk group for COVID-19 disease, an attitude of absence was experienced in the offices for the preservation of these individuals. Conversely, some urgent routines became frequent; consequently, the dental office became more frequented.

*“I saw a severe reduction of treatments, a severe reduction of treatments.”*

(Surgeon 1)

*“…negative questions, everybody is more afraid, so the flow of patients has decreased, older people who I think are the largest part of my patients today (…). Recently, they are the people who are most afraid to leave home and to do anything, and with plenty of reason, you know?!”*

(Surgeon 6)

*“…I spent almost one year without working (slightly changes the tone of her voice) due to the pandemic. First of all because nobody knew what they were dealing with.”*

(Surgeon 4)

*“…I think I reduced the demand for care a lot. For surgery, I think this is it. Postponing things that we could do today and the prevention of problems for the patients.”*

(Surgeon 8)

In contrast, some participants reported that there was no reduction in attendance and, on the contrary, in some places in the city, the pandemic did not change the local field of work at all.

*“Here in my region, there never was isolation. The police passes by and doesn’t do anything (shows an expression of disbelief and shakes his head). So, my office and my practice were not affected.”*

(Surgeon 9)

*“So, my movement increased a lot (raises his eyebrows), my demand hit the ceiling and I never had to stop working for a single day during the pandemic.”*

(Surgeon 7)

#### Urgent care during the COVID-19 pandemic

As a result of exacerbated levels of stress and anxiety, a new wave of emergency care was performed in dental offices. This created a social perception of the need for an integrated team present in the public and private sectors, including hospitals.

*“…The clinic was practically almost ready to satisfy this demand (raises his eyebrows) and considering that the patients mainly needed urgent care. Feeling pain with pulpitis or even trauma.”*

(Surgeon 7)

*“…where I work in the SUS (Brazil’s Unified Health System) I have not had many elective surgeries, you know. I practically did only urgent surgeries.”*

(Surgeon 8)

*“…specifically, regarding temporomandibular dysfunction, there was an explosion of cases (a change in his tone of voice and in his countenance, and emphasis on his reply) during the pandemic. I treated, on average, two patients a week, patients who reported pain in the joint, tooth lock, headache (initially he makes a face of astonishment and then completes the sentences with his eyes closed).”*

(Surgeon 7)

#### Patients afraid to be treated

Due to the fear of an as-yet-unknown disease, with a brief worldwide history of high mortality rates, adults became averse to leaving their home protection, developing, in some cases, fear of dental treatment.

*“…because the patients were greatly afraid to be submitted to dental care.”*

(Surgeon 2)

*“…the fear people had to look for care during dental visits, the fear to be contaminated, the fear to be greatly exposed, because the patients wear no mask…”*

(Surgeon 8)

*“During this time, the hospital environment became an environment of fear for people. People don’t want to go to the hospital because they are afraid to catch COVID there (she talks more calmly at this time and practically does not gesticulate)! And even though this is an environment 100% sterilized, with a very high sanitary surveillance. You may get infected there or in other environments, but the hospital environment has been stigmatized as a high-risk place!”*

(Surgeon 4)

#### Financial commitment

Associated with the aforementioned fact of suspension of care and its subsequent spacing, another factor that impacted the financial loss of professionals participating in the study was the fear shown by patients to continue or start treatment, whether in an outpatient or hospital setting. Another factor that contributed to this, according to one of the participants, was the financial difficulty that the patients themselves also began to have.

*“…the patients, also simply due to financial questions that are equally affected, are postponing treatment, so there definitely was an impact also on our professional profitability.”*

(Surgeon 2)

*“It really was quite reduced and it affected the class economically, it affected everybody (shows slight emphasis in his speech) and we had to adapt…”*

(Surgeon 1)

*“…our financial part was extremely impacted (he now shows concern in addition to frowning) due to the lack of daily service.”*

(Surgeon 5)

*“…the fear to catch COVID, the fear to transmit this COVID, okay? And the general panic that this generated for the patients and the consequent economic impact on the activity (emphasizes his speech with a positive head shake).”*

(Surgeon 3)

*“And I must say that the negative part was the price of the materials, which became very expensive! Very expensive!!”*

(Surgeon 7)

**Fig 1:**
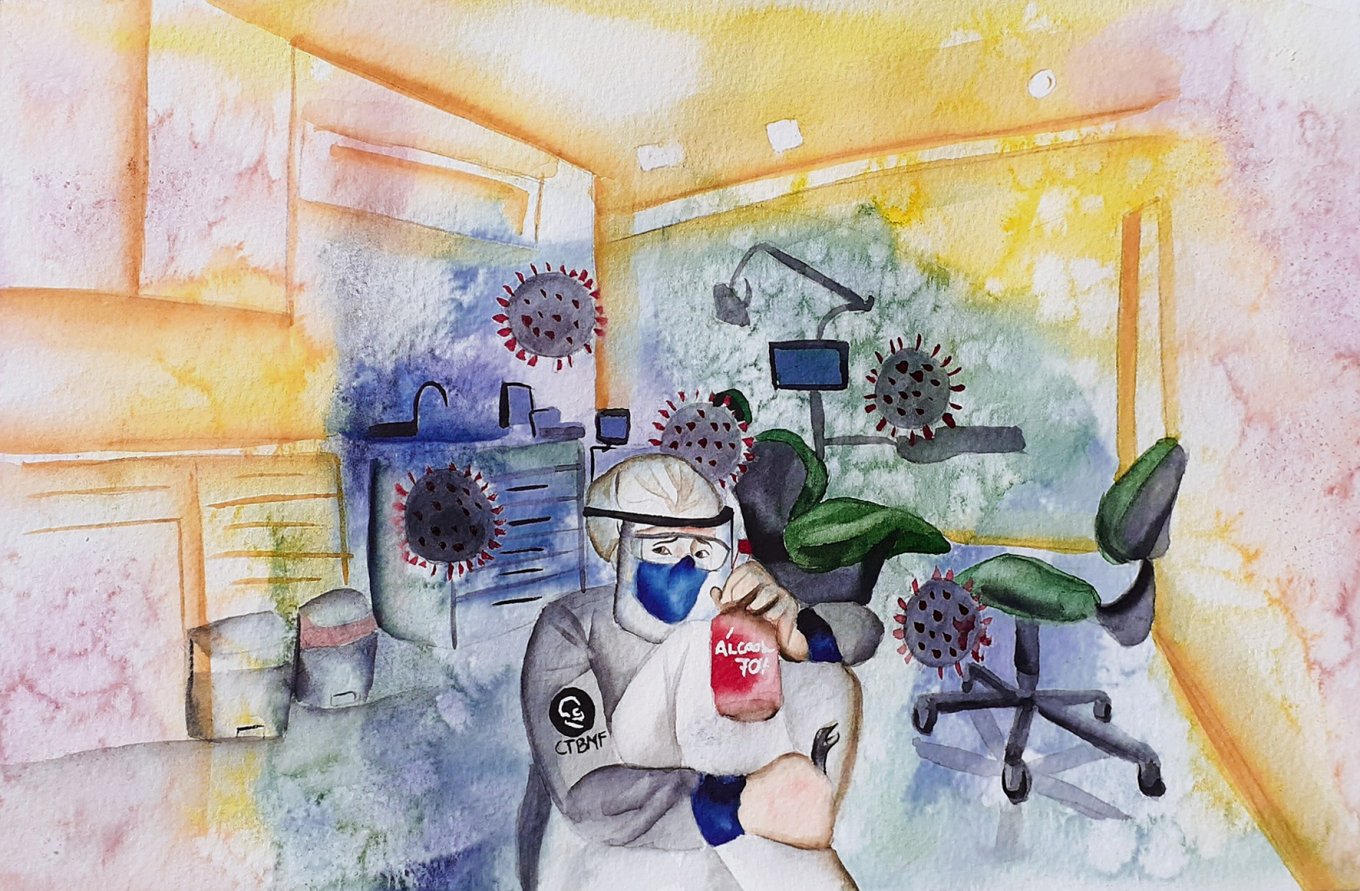
Representativeness of the feeling of stress experienced by oral and maxillofacial surgeons in the dental office and/or hospital setting.

Most participants reported a direct relationship between the initial interruption of attendance and its reduction due to the declaration of a pandemic by the World Health Organization and the decrease in earnings in their work environments, projecting a large financial impact on their lives. These data revealed an increase in anxiety, as illustrated in **Fig 1**.

## Discussion

The present qualitative study demonstrated for the first time a deeper relationship between the experiences of oral and maxillofacial surgeons and the COVID-19 pandemic. Moreover, this study documented the memories and feelings of the participants, revealing the perception of behavior in light of society’s reflection on fear, risk of contagion, stress, mental fatigue, and comfort.

The notes and reports most frequently pointed out in the study concerned the fear of a still unknown universe, of SARS-CoV-2 and its form of action, which was responsible for a worldwide spread with thousands of people affected and deaths. With the effect of globalization and the ease of intercontinental travel, the dissemination of COVID-19 has interrupted the social, economic and emotional well-being of our society, reflecting on all spheres [1]. As expected, the initial effects reported by the respondents were ignorance and fear as the spread of the virus exceeded the way biosafety controls and protocols worked and initially required absence from daily work. In fact, biosafety standards were the target of public policies in Brazil in the past; however, studies have pointed out non-compliance with these standards by portions of the population of dentists [16,17].

In the experience of the participants, the fear of being contaminated during care or in the workplace was mentioned with great frequency, both in the assessment of the public sector and of the private sector (hospital outpatient clinic or dental office). New regulated resolutions from the Brazilian Health Regulatory Agency (Anvisa) and Brazilian Federal Council of Dentistry (CFO) have made public recommendations for care during the COVID-19 pandemic. Questionnaires about possible contacts with COVID-19 that patients may have had as well as guidance on the correct use of personal protection equipment were some of the items [18,19]. However, there was a need for oral health professionals to tailor their activity to the new guidelines for greater safety, for both the patients and their caregivers. In particular, the specialty of oral and maxillofacial surgery represented an example of the need to adapt considerably to this outbreak, especially in the hospital setting [7]. Due to the lack of knowledge about the risks and the fear of being contaminated during work, authorities proposed measures that interfered with the pace of care, initially causing a total interruption. But, according to the reports of the respondents, work was resumed for urgent and emergency care, with elective surgical procedures being relegated to the background.

In the current study, most participants pointed out an increase in biosecurity with more rigorous and beneficial control measures such as the wear of appropriate personal protection equipment, especially N95 and PFF2 masks, in addition to the use of face shields. The safest management of patient admission consisted of measures adopted to reduce anxiety levels and to encourage the return to routine care [20]. Individuals also reported that there was a disturbing change in their anxiety levels, with only one of them stating that he/she did not perceive a negative influence on this aspect as an effect of the pandemic. Notably, the present findings are similar to those reported in a study on residents of oral and maxillofacial surgery in the United States, where all professionals had changes in anxiety levels, especially women and senior surgeons [6]. Another study showed that training programs in oral and maxillofacial surgery adopted measures to better protect their professional teams in order to reduce stress and maintain well-being [21]. This fact was also observed herein, in which we noticed that the protective measures adopted regarding a stricter biosafety protocol and greater spacing between appointments managed to reduce the professionals’ levels of stress and anxiety.

Most participants in this investigation mentioned that the determination to reduce or even discontinue activities at the beginning of the COVID-19 pandemic provoked a change in their work routine, reflecting significantly and negatively on their finances. This is in line with a previous study in which the reduction in patient care and the implementation of restrictive preventive measures created financial difficulties for most dental offices [22]. We also noticed that, due to one of the recommendations proposed by the authorities as a containment measure, there was an increase in the spacing between consultations, as well as the impossibility of maintaining the health insurance routine, consequently generating financial inflow. According to Ferneini [23], oral and maxillofacial surgeons are already facing a financial crisis with consequent economic deterioration. Furthermore, as the COVID-19 pandemic begins to subside, many surgeons may not be able to restart their practice, becoming aware of the financial impact on their practice in the short- and long-term [24].

In this respect, the decrease in purchasing power was projected by the participants as yet another factor triggering stress and anxiety levels. In addition, despite this break in the work setting during the onset of the pandemic, a certain level of stress was still reported, in contrast to the study of dentists in Turkey who continued to work during the COVID-19 pandemic, and this was significantly related to occupational burnout, high levels of stress, and psychological exhaustion among those professionals [25]. Still regarding the psychological effect of the pandemic, our participants pointed out medium to high changes in the rates of mental exhaustion capable of significantly affecting their routines, as also documented elsewhere [6]. Indeed, the high levels of anxiety and stress experienced by health professionals during the period of the pandemic was also observed in other studies, demonstrating the need to implement policies to combat the mental strain of this target audience [6,21].

As reported frequently by the participants of the present study, the fear of being contaminated in the work environment despite the management measures and protective barriers, and of secondarily contaminating their family members, especially older adults or individuals belonging to risk groups, mainly their parents, was the most frequent reason for isolation, change in lifestyle, habits, or even the need for a permanent change of address. Overall, there has been a direct influence of the COVID-19 pandemic on oral health professionals, with behavioral changes, increased levels of stress and a personal and social impact since its inception, particularly in Latin America and the Caribbean [26]. This aspect was also reported by respondents as a reason for increased daily stress and anxiety levels and for generating changes not only in the interaction with family members, but also in terms of the need to deal differently with daily household routines regarding the handling of food and the need to create secondary biosafety protocols when handling the clothes used at work in their homes.

The study has some limitations such as the time elapsed between the memories of the respondents and the time of their interviews. Nevertheless, the variation in age, time since graduation and work environment provided a sample capable of reproducing cohesive memories, enough to create emotions in our participants. A fact that should be highlighted is that the researchers involved in this study were dentists. Despite this, we believe that capturing the details and feelings of respondents by dentist-interviewers was more accurate and reliable, especially in the context of data interpretation. However, possible judgments of the interviewer–even if unconscious–cannot be completely ruled out. Thus, a possible strategy, for future studies would be the involvement of a multidisciplinary team in the study design. Conversely, we were able to obtain an in-depth and relevant profile of the experience lived by oral and maxillofacial surgeons during this moment of adversity of the COVID-19 pandemic that will remain forever imprinted. As future directions, we strongly encourage carrying out qualitative studies with the purpose of better interpreting how facts and acts affect the population’s psyche, as well as their comfort and personal and professional well-being. Finally, it is worth mentioning that all participants showed psychological willingness to participate in this study.

## Conclusions

In summary, after the advent of COVID-19, a series of developments had repercussions on the personal-professional axis of oral and maxillofacial surgeons. Consistently, there has been a complete change in their daily work routines, both in the hospital and outpatient setting, regardless of the public or private sector. Such modifications occurred due to the need to change existing protocols, which at the time proved to be ineffective in coping with COVID-19.

The pandemic had a direct impact on work activities, with financial losses for these professionals. Likewise, the lockdown also contributed to changing the social relationship of this public. With the gradual return to work with new biosafety guidelines, it culminated in the need for these professionals to be absent from their home, family and friendship cycles. This fact had psychological repercussions since these professionals were considered possible vectors. As such, increased levels of stress and anxiety were recorded by the oral and maxillofacial surgeons.
